# Co‐production of health and social science research with vulnerable children and young people: A rapid review

**DOI:** 10.1111/hex.13991

**Published:** 2024-02-25

**Authors:** Jo Erwin, Lorna Burns, Urshla Devalia, Robert Witton, Jill Shawe, Hannah Wheat, Nick Axford, Janine Doughty, Sarah Kaddour, Abigail Nelder, Paul Brocklehurst, Skye Boswell, Martha Paisi

**Affiliations:** ^1^ Peninsula Dental School University of Plymouth Plymouth UK; ^2^ Royal National ENT and Eastman Dental Hospital London UK; ^3^ School of Dentistry Royal Liverpool University Dental Hospital Liverpool UK; ^4^ NHS England Leeds UK; ^5^ Peninsula Dental Social Enterprise Truro UK; ^6^ Public Health Wales Cardiff UK; ^7^ School of Nursing and Midwifery University of Plymouth Plymouth UK

**Keywords:** children and young people, co‐production, rapid review, vulnerable

## Abstract

**Background:**

The term ‘care‐experienced’ refers to anyone who is currently in care or has been in care at any stage in their life. A complex interplay of factors leads to care‐experienced children and young people (CECYP) experiencing poorer oral health and access to dental care than their peers. A rapid review of the co‐production of health and social care research with vulnerable children and young people (CYP) was carried out to inform the development of a co‐produced research project exploring the oral health behaviours and access to dental services of CECYP. Here, ‘co‐production’ refers to the involvement of CYP in the planning or conduct of research with explicit roles in which they generate ideas, evidence and research outputs.

**Aim:**

To learn how to meaningfully involve vulnerable CYP in the co‐production of health and social science research.

**Objectives:**

To identify: Different approaches to facilitating the engagement of vulnerable CYP in co‐production of health and social science research; different activities carried out in such approaches, challenges to engaging vulnerable CYP in co‐production of health and social science research and ways to overcome them and areas of best practice in relation to research co‐production with vulnerable CYP.

**Search Strategy:**

A rapid review of peer‐reviewed articles was conducted in six databases (MEDLINE, Embase, SocINDEX, CINAHL, PsycINFO and Web of Science) and grey literature to identify studies that engaged vulnerable CYP in co‐approaches to health and social research.

**Main Results:**

Of 1394 documents identified in the search, 40 were included and analysed. A number of different approaches to co‐production were used in the studies. The CYP was involved in a range of activities, chiefly the development of data collection tools, data collection and dissemination. Individual challenges for CYP and researchers, practical and institutional factors and ethical considerations impacted the success of co‐production.

**Discussion and Conclusion:**

Co‐production of health and social science with vulnerable CYP presents challenges to researchers and CYP calling for all to demonstrate reflexivity and awareness of biases, strengths and limitations. Used appropriately and well, co‐production offers benefits to researchers and CYP and can contribute to research that reflects the needs of vulnerable CYP. Adherence to the key principles of inclusion, safeguarding, respect and well‐being facilitates this approach.

**Patient and Public Contribution:**

Members of our patient and public involvement and stakeholder groups contributed to the interpretation of the review findings. This manuscript was written together with a young care leaver, Skye Boswell, who is one of the authors. She contributed to the preparation of the manuscript, reviewing the findings and their interpretation.

## 1 INTRODUCTION

Inequalities in oral health relate to social determinants of health, including poverty and social exclusion.^1^ Due to the complex interplay of a range of factors care‐experienced children and young people (CYP) experience worse oral health than their peers. Those in care are more likely to come from lower‐income families, to have experienced neglect and to have experienced significant transitions (e.g., care placements in different geographic locations) which can disrupt oral health service continuity. While oral health problems and access to dental services for these CYP have been identified as in need of attention,^2^ the research needed to inform policy and practice is limited.

To address this gap in the evidence, a study (‘Dental care for CECYP—Caring for children and their smiles’) is being undertaken by the authors to explore and provide evidence of the oral health behaviours, dental experience and access to dental services of care‐experienced children and young people (CECYP). A key aspiration of this study of oral health inequalities in this often‐marginalised group is to work collaboratively with CECYP to co‐produce the research. Co‐production acknowledges that people with ‘lived experience are often best placed to advise on what support and services will make a positive difference to their lives’.^3^ It was deemed especially important to build co‐production into this project due to CYP in care and care leavers belonging to one of the most marginalised groups in society^4^ who frequently do not have the opportunity to have their voices heard.

To ensure co‐production was effective and a positive experience for those involved, we considered it important to learn how others have meaningfully involved vulnerable CYP in the co‐production of research. We chose a rapid review to assess what is known about co‐production with vulnerable CYP because it allowed us to systematically search the existing research in a timely and resource‐efficient way consistent with the timeframe of the wider project.^5^ Rapid reviews are recognised as a legitimate method and have been used increasingly in recent years.^6^ It is a type of evidence synthesis that follows robust, systematic approaches but also allows for a shortened timescale.^7^

## 2 CO‐APPROACHES AND PARTICIPATORY RESEARCH

The terms co‐creation, co‐design and co‐production are often used interchangeably and are ill‐defined.^8^, ^9^ They all refer to some extent of active participation of stakeholders and/or end users in the research process and an acknowledgement of the knowledge that arises from lived experience. ‘Co‐creation’ is often used to refer to approaches that are systems‐based and focused on innovation.^10^, ^11^ Co‐design has been defined as ‘meaningful end‐user engagement in research design’^12^, ^pp.2–3^ with engagement ranging from being relatively passive to highly involved in all stages of the research process.

‘Co‐production’ refers to more than ‘meaningful end‐user engagement’. It is committed to working in partnership to generate ideas, evidence and research outputs, recognising the importance and validity of different forms of knowledge. There are a number of approaches in the ‘family’ of participatory research. These include community‐based participatory research (CBPR),^13^ youth participatory action research (YPAR),^14^ participatory action research (PAR)^15^ and peer research.^16^ In CBPR there is an emphasis on engagement with the community and social justice. Community members define the problem to be explored, helping researchers navigate the social and cultural milieu of the community and acting as advisors (or taking more active roles) throughout the project.^13^ YPAR is a participatory approach focussed on positive youth and community development, which is action‐oriented. There is an emphasis on skills development and the positive contribution it can make to improving individuals' lives and that of their communities.^14^ Peer research explicitly enables members of the population group to whom the research relates to and who have lived experience, to take on an active role in directing and conducting research.^16^ Other approaches that involve CYP in research draw on their knowledge and experience, including Patient and Public Involvement and Engagement (PPI/E) groups^17^ and Young Persons' Advisory Groups (YPAGs).^18^ PPI/E groups are used widely by researchers to provide the perspective of young people. In health service organisations, the role of these groups is to represent the interests of CYP in the development of services, guidance and quality standards.^19^ YPAGs provide advice on research involving CYP. Researchers in health disciplines can take their research proposals to a YPAG for scrutiny and advice. YPAGs often consider the wider impacts of the research topic and study design on participants.^18^

Given the lack of clarity around definitions, we took a pragmatic approach to the rapid review and chose to view ‘co‐production’ as encompassing co‐creation, co‐design, co‐production and participatory approaches. Informed by Slattery et al.,^12^, p.3 we take the definition of co‐produced research as ‘involvement of CYP in an explicitly described role contributing to the planning and/or conduct of [health] research’. This includes all aspects and stages of research from the identification of research priorities through to dissemination of results. We will use the term ‘co‐production’ throughout this article to encompass the approaches that meet this definition.

‘Vulnerable children’ can be defined as ‘any children at greater risk of experiencing physical or emotional harm and/or experiencing poor outcomes because of one or more factors in their lives’.^5^ Examples of children who are identified as vulnerable include those that: have safeguarding concerns or are in state care; have health problems disabilities; health or developmental problems; are low income; have challenging family circumstances; are not engaged in or excluded from education; are involved in offending or antisocial behaviour and have experience of abuse/exploitation or come from minority populations.^6^

### 2.1 Aim

To learn how to meaningfully involve vulnerable CYP in the co‐production of health and social science research.

### 2.2 Objectives

1. To identify different approaches to facilitating the engagement of vulnerable CYP in the co‐production of health and social science research.
2. To identify different activities carried out in such approaches.
3. To identify challenges to engaging vulnerable CYP in the co‐production of health and social science research and how these have been overcome.
4. To identify areas of best practice in relation to research co‐production with vulnerable CYP.

## 3 METHODS

### 3.1 Search of electronic databases

Searches of the peer‐reviewed and grey literature were carried out to identify studies that have engaged CYP in co‐approaches to health and social research. The following health and care databases were searched:

#### 3.1.1 MEDLINE, Embase, SocINDEX, CINAHL, PsycINFO and Web of Science

The searches were designed and undertaken by an information specialist (L. B.) following consultation with the team. There were three blocks of terms to represent the concepts of children or young people; characteristics of vulnerability and co‐production. The search histories are detailed in Data S1.

An additional search for grey literature was carried out. The sites searched were:

Google, EThOS, the Health Foundation, Social Care Online, ClinicalTrials.gov, Fostering Network, Voice of the Child in Care, NSPCC and Who Cares Trust, Safeguarding network, Early Intervention Foundation, Barnardo's, INVOLVE, Health Systems Evidence and James Lind Alliance.

The searches were carried out in September 2022 and results were downloaded to and duplicated in EndNote. References were sorted and selected for screening against the eligibility criteria using Rayyan.^20^ One reviewer (J. E.) screened titles and abstracts. The full texts were obtained and screened by the reviewer (J. E.) using the eligibility criteria. In cases of doubt, a second reviewer (M. P.) was consulted and a consensus was reached.

### 3.2 Study selection and eligibility criteria

Studies were selected using the following eligibility criteria:

Publications were sought that related to the co‐production of health and social science research by vulnerable CYP aged up to 25 years old. Eligible publications were written in English. Any study design such as randomised controlled clinical trial, nonrandomised trial, cohort study, pilot study, feasibility analysis, single case design, survey and qualitative investigations were eligible for inclusion. No date restrictions were applied.

Studies relating solely to the participation of vulnerable CYP in the testing of health technologies or clinical studies were excluded. In addition, brief articles, conference abstracts, commentaries, letters, medical newsletters, book reviews, protocols, book chapters, editorials and conference abstracts without an accompanying paper were excluded.

### 3.3 Data extraction and quality assessment

One reviewer carried out the data extraction (J. E.) using a data collection form to support the search strategy. The data collection form was created to identify the key elements of included studies needed to answer the review questions. This included aims/objective, study design, country, vulnerability of CYP, age of participants, co‐production approach, number actively involved in co‐production, activities, challenges to co‐production and how challenges to co‐production were overcome. This structured approach supported the compilation of consistent information from a variety of study designs. Reference and publication information about each study were also collected (e.g., authors, title, publication date).

A large number of articles were reflections on, rather than descriptions of, qualitative research and did not describe in detail the research methods. Based on this, and the time restraints associated with a rapid review, the decision was taken not to carry out a quality assessment of the papers included in the review.

### 3.4 Data analysis

The publications identified in the search were categorised as:

1. Descriptive papers—peer‐reviewed articles and reports describing the methods and results of health and social science research studies co‐produced with vulnerable CYP.
2. Reflective papers—peer‐reviewed articles and reports describing and reflecting on lessons learnt and challenges identified in the process of co‐producing health and social science research with vulnerable CYP.
3. Review papers—scoping or systematic reviews of studies or projects using co‐production with vulnerable CYP to conduct health and social science research.

The findings from the included articles were synthesised using a qualitative descriptive approach.^7^ Descriptive summaries of the findings relating to the key elements (co‐production approach, activities, challenges to co‐production, how challenges were overcome were made) were created. Similar themes identified in the summaries were collated into ‘topics’. This allowed the findings of the studies/reports to be summarised and grouped. This process was carried out by J. E. and H. W.

## 4 RESULTS

**Figure 1:**
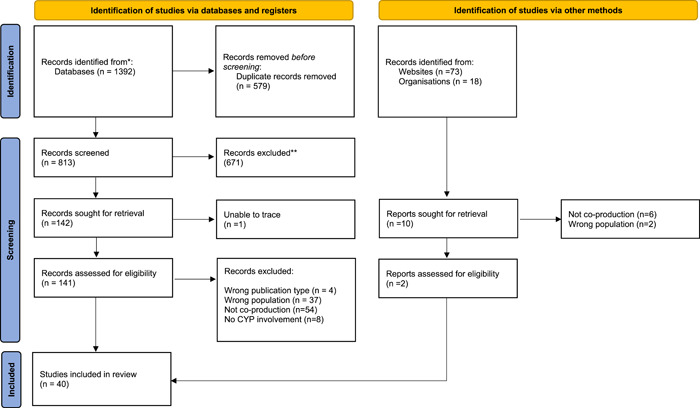
PRISM diagram.

A total of 1392 articles and reports were identified in the search, of which 38 met the inclusion criteria. An additional two publications (one toolkit and one review) were identified and included in this rapid review (for details see Figure 1). As defined above, the following types of publication were identified in the search: descriptive (*n* = 16), reflective (*n* = 20) and review (*n* = 5).

Table 1 shows the characteristics of the descriptive and reflective studies included in this review. Please note that the information on these characteristics was missing in some studies.

**Table 1:** Study characteristics.

Characteristic	Description, (number of studies)
Origin of study	UK (14) North America (11) Africa (5) Europe (3) Middle East (1)
co‐production approach	Participatory/PAR (11) Peer research (7) CBPR (4) PPI/E (4) Co‐production (3) YPAR (2) YPAG (2)
Vulnerability of CYP	Disabled (7) Children in care/Care leavers (5) Homeless (5) Street connected (5) Mental health (3) Health (2) Neurodiverse (2) Low income (2) LGBTQ (1) Refugee (1)
Age range of CYP	Range 3–25 years old
Range of number of CYP co‐researchers	Range 2–36 <5 = 9 5–9 = 7 10–14 = 8 15–20+ = 8

Abbreviations: CBPR, community‐based participatory research; CYP, children and young people; LGBTQ, lesbian, gay, bisexual, transgender, and queer; PAR, participatory action research; PPI/E, Patient and Public Involvement and Engagement; YPAG, Young Persons' Advisory Groups; YPAR, youth participatory action research.

The studies were mainly conducted in the UK and North America. The studies were categorised by their authors as participatory, PAR, CBPR, YPAR, peer research, co‐production, YPAG and PPI/E. The most commonly used methods were participatory (including PAR) and peer research. The CYP most frequently involved were those with disabilities, CECYP, homeless or street connected (a CYP who spends a portion or a majority of his or her time living or working on the streets). The mean age range of the CYP was 14.7–21.3 years. In nearly 50% of studies (where data was available), the minimum age of the CYP involved was 16.

More details of the studies included in the review can be found in Data S2.

The topics identified are described below:

### 4.1 Topic 1: Activities carried out in the co‐production of health and social science research with vulnerable CYP

#### 4.1.1 Types of activity

The CYP were involved in a range of activities in the process of co‐production. Table 2 presents the different activities carried out by CYP categorised by the co‐production approach (descriptive papers and reflective papers only).

**Table 2:** Co‐production approach and associated activities.

Activity	Peer research	CBPR	YPAR	Participatory	YPAG	PPI/E	Co‐production
Identify research priorities				Afifi et al.,^21^ Dadswell et al.,^22^ Gray et al.^23^	Pavarini et al.,^18^ Sime et al.^24^	Mawn et al.,^25^ Morris et al.^26^	
Identify research questions	Brady et al.^27^	Lincoln et al.^28^		Varjavandi^29^			
Contribute to the choice of research design/method		Mitchell et al.^30^	Ritterbusch et al.^31^	Gray et al.,^23^ Nichols and Malenfant^32^	Pavarini et al.^18^	Alderson et al.^33^	Liddiard et al.^34^
Develop data collection tools	Kelly et al.,^35^ Noom et al.,^36^ Taylor et al.,^37^ Torronen et al.^38^	Garcia et al.^39^ Thulien et al.^40^	Hillier et al.,^41^ Ritterbusch et al.^31^	Dadswell et al.,^22^ Embleton et al.,^42^ Funk et al.,^43^ Lam et al.,^44^ Varjavandi,^29^ van Staa et al.^45^			Liddiard et al.^34^
Recruitment		Lincoln et al.^28^	Ritterbusch et al.^31^				Liddiard et al.^34^
Data collection	Brady et al.,^27^ Curran et al.,^46^ Kelly et al.,^35^	Garcia et al.,^39^ Lincoln et al.,^28^ Mitchell et al.,^30^	Hillier et al.,^41^ Ritterbusch et al.,^31^	Coser et al.,^47^ Gray et al.,^23^ Kramer et al.,^48^ Lam et al.,^44^ Nichols and Malenfant,^32^ Varjavandi,^29^ van Staa et al.^45^			Chappell et al.,^49^ Liddiard et al.^34^
Data analysis/interpretation	Burke et al.,^50^ Curran et al.,^46^ Taylor et al.,^37^ Torronen et al.,^38^	Garcia et al.,^39^ Lincoln et al.,^28^ Mitchell et al.^30^	Ritterbusch et al.^31^	Coser et al.,^47^ Dadswell et al.,^22^ Funk et al.,^43^ Kramer et al.,^48^ Lam et al.,^44^ Varjavandi^29^			Chappell et al.,^49^ Damian et al.^51^
Dissemination	Burke et al.,^50^ Torronen et al.,^38^	Lincoln et al.,^28^ Mitchell et al.,^30^ Thulien et al.^40^	Hillier et al.,^41^ Ritterbusch et al.^31^	Coser et al.,^47^ Dadswell et al.,^22^ Funk et al.,^43^ Gray et al.,^23^ van Staa et al.^45^	Pavarini et al.^18^	Mawn et al.^25^	Liddiard et al.^34^

Abbreviations: CBPR, community‐based participatory research; PPI/E, Patient and Public Involvement and Engagement; YPAG, Young Persons' Advisory Groups; YPAR, youth participatory action research.

CYP were most often involved in the development of data collection tools, data collection and dissemination, less often in the identification of research ideas or priorities. The CYP were active in the reviewing and development of interview and focus group topic guides, testing them for face validity and appropriateness of language. They participated in data collection, including administering surveys, using photovoice (a qualitative research method that gathers participant‐taken photographs and narratives to translate experience into knowledge), conducting interviews and facilitating focus groups. In a number of studies, CYP were involved in the analysis and interpretation of qualitative data but in general, few details were given of the extent or the nature of their role. Dissemination activities in which CYP participated included report writing, co‐authoring academic papers, producing blogs, podcasts and videos, holding public engagement events and knowledge translation activities and presenting at conferences. In two studies,^18^, ^33^ CYP developed resources to raise awareness of research in CYP and to encourage their involvement in research.

#### 4.1.2 Role of CYP

In all studies, the CYP had an explicit role of providing the perspective of CYP. While CYP were generally active in a wide range of roles, in only three studies^22^, ^23^, ^28^ were they active across the whole research process from research priority/question identification through to dissemination of findings. This supports Flotten et al.'s^20^ observation that few co‐produced studies include CYP researchers at every stage.

#### 4.1.3 Types of incentives for CYP involvement

Eleven studies^18^, ^26^, ^28^, ^33^, ^34^, ^36^, ^38^, ^41^, ^45^, ^46^, ^47^ reported on the incentives given to the CYP co‐researchers. In all but one of the studies^34^ the incentives were financial, in the form of money or vouchers. In three studies co‐researchers received university certificates and references.^34^, ^38^, ^46^ In three of the UK studies,^18^, ^33^, ^34^ the authors cited the NHS INVOLVE guidance on reward and recognition for CYP involved in research^52^ when reporting on the incentives given. No other studies indicated how they decided the type and level of incentive given to co‐researchers.

### 4.2 Topic 2: Individual challenges for CYP engaging in co‐production and how to overcome them

#### 4.2.1 Barriers/facilitators to CYP's engagement with co‐production arising from the complexity of their lives

CYP have complex and dynamic lives^25^ and therefore have to balance co‐production with their education, social, work, sport and family commitments. For vulnerable CYP, taking an active role poses even greater challenges—for example, owing to disabilities, health problems, shifting life circumstances or limited resources, or because they are in care, homeless or street connected.^18^, ^34^, ^38^, ^40^, ^45^, ^51^

Working with and accommodating the needs of vulnerable CYP can necessitate providing support to CYP across many aspects of their daily lives.^45^, ^47^ Building mutual trust and understanding and supporting CYP to engage through difficult times is important.^43^, ^47^ Training, team‐building activities and regular meetings can help build group cohesion.^43^, ^47^, ^53^ It is important to have an environment in which CYP are comfortable, with refreshments and frequent breaks.^33^, ^40^, ^47^, ^54^ Practical measures such as providing transport to sessions are helpful.^33^ Flexibility around the location, timing and type of interaction is needed to make it as easy as possible for CYP to be involved in co‐production.

#### 4.2.2 Barriers/facilitators to maintaining motivation/interest

Establishing and maintaining interest and motivation in the research can be a challenge.^25^ In projects that can last months or years this can mean that CYP will come and go from the group, necessitating on‐going recruitment, which has implications for continuity and group dynamics.^54^ Making the research process interactive and task‐driven^33^, ^55^ and identifying motivators that can facilitate engagement with the research co‐production process can help maintain interest.

#### 4.2.3 Barriers and facilitators to CYP acquiring new skills that enable involvement in research

Researchers found that despite trying to accommodate their needs it was not always feasible to fully involve the CYP in all aspects of their project,^43^, ^47^, ^50^ partly because there was a lack of time to teach research techniques but also owing to CYP's varied abilities.^43^, ^44^, ^50^ It is important to affirm CYP's existing expertise while supporting the development of new skills and confidence,^40^ recognising and adapting training and participation to the abilities and capacity of the CYP.^18^, ^43^, ^47^, ^50^

### 4.3 Topic 3: Challenges for adult researchers

#### 4.3.1 Discomfort due to lack of experience

Choosing to co‐produce research with vulnerable CYP necessitates adult researchers taking on a participatory approach which they may not be familiar with. Building relationships with CYP and supporting them in co‐production may involve the adult researchers working in different ways, which can bring additional responsibilities, such as supporting CYP practically and emotionally during the process.^38^, ^47^

Adult researchers need resilience, patience and tolerance^21^, ^33^ to work with vulnerable CYP. Dovey‐Pearce et al. described how adult researchers can sometimes feel anxious about how best to involve CYP.^55^ Torronen and Vornanen^38^ described how some of the CYP's ideas were challenging for adult researchers. Adults sometimes needed to be reminded to listen to the CYP, to respect them, their culture and what they bring to the table. Adults can play an important positive role in situations where CYP may lack acceptance or stability in their daily lives.^39^ Investing time in getting to know the CYP and their community and understanding the context of their lives can help foster mutual understanding and trust.

#### 4.3.2 Changing existing power dynamics and hierarchy

It can be difficult for adult researchers to relinquish power to the CYP with whom they are co‐producing research. Issues of power and hierarchy were identified in a number of studies.^39^, ^40^, ^41^, ^49^ Bringing CYP and adults together early in the research process,^39^ collaborative decision making and shared leadership of the research processes can help with power relationships.^40^, ^41^ In relation to her work with disabled CYP, Brady and Franklin^27^, p.8 referred to ‘the balancing act within the research study between the young disabled researchers becoming leaders and decision‐makers but subject to imposed institutional and procedural constraints’. Liddiard et al.^28^ point to the fact that disabled young women are often shut out of leadership roles and that thoughtful accessible modes of co‐production can open up such opportunities.

Working with a refugee community, Afifi et al.^21^ found that where communities are patriarchal, cultural norms may prevent youth from speaking out in front of adults, especially when they disagree. This problem can be circumnavigated by the youth creating their own committee with a representative attending the main (adult) committee meeting.

### 4.4 Topic 4: Inclusivity

#### 4.4.1 Lack of diversity in co‐produced research

Researchers are often motivated to use participatory methods because they explicitly support the inclusion in research of those who may be marginalised and excluded.^9^ Peer research is highlighted as an important method for supporting inclusivity.^55^ However, Liddiard et al.^34^ observe a lack of diversity in co‐produced research with disabled CYP. Bradbury Jones et al.^9^ suggest that adults may doubt the competence of younger CYP and those who do not have advanced communication skills. The lack of co‐produced research with younger children is evident from this review, with only two of the studies,^23^, ^24^ including children aged 10 or under. Bailey et al.^56^ identified the lack of involvement of CYP with non‐verbal communication or complex impairments and those from minority ethnic groups.

### 4.5 Topic 5: Practical barriers to co‐production

#### 4.5.1 The financial cost of flexibility

A challenge to co‐production raised in a number of studies^31^, ^43^, ^47^, ^54^, ^56^ was cost. Co‐production of research requires flexibility to respond to the varying needs and abilities of CYP to help CYP overcome personal barriers to participation and to support them in difficult times.^47^ This can have significant cost implications. As the involvement of CYP in co‐production is often a fluid and evolving process,^33^ there may be a degree of unpredictability about the cost. Funk et al.^43^ described how, to accommodate increasing participation and the differential learning of CYP, extra training sessions were created and deadlines extended, all of which had budget implications.

#### 4.5.2 The financial costs associated with building positive relationships between adult researchers and CYP

There are costs associated with developing positive relations with CYP co‐researchers, for instance hiring youth friendly venues, travel, refreshments, activities and training.^54^ Participatory research requires trust and capacity building of both academics and CYP, which is more time‐consuming than having an ‘expert’ develop and implement a research project.^21^ Coser et al.^47^ and Ritterbusch et al.^31^ highlight the need for project budgets to be realistic and to allocate money for the professional development and acknowledgement of co‐researchers.

### 4.6 Topic 6: Institutional challenges

#### 4.6.1 The nature of research funding

As funding is usually tied to a particular area of study or agenda, it can be difficult for CYP to set the agenda or research questions. This negatively impacts their ability to engage with the initial formative stages of a research project.^57^ There is a lack of funding for ongoing PPI/E initiatives compared with one–off research projects^25^ and the fluidity of activities associated with co‐producing research with vulnerable CYP may not sit well with the needs of funders.

#### 4.6.2 The role of gatekeepers

Sime^24^ highlighted the challenges associated with the practicalities of gaining access to children through gatekeepers at various institutions, such as schools and children's clubs, and the need to convince them of the research's worth. In a study of care leavers, the mixed public/private nature of the Finnish child welfare system meant that it was impossible to obtain contact information for young people who had left care.^38^

#### 4.6.3 Negotiating academic conventions about co‐authorship

Hillier and Krorehle^41^ described how their YPAR collaboration pushed up against established conventions about co‐authorship in an academic paper and what co‐authorship entails. Co‐authoring with the CYP involved more time and negotiation around who makes revisions and how they are made than the faculty member's typical experience of co‐authorship.

#### 4.6.4 Expectations of research managers

Dovey‐Pearce et al.^55^ described the tension arising from the realities of running co‐production research, with managers wanting work done at pace and feeling the project was being ‘slowed down’ by the young people's involvement. Members of the management group were invited to some of the early co‐production sessions to help them understand the process, why more time was needed and the benefits to the project of giving that time.

### 4.7 Topic 7: Ethical considerations

#### 4.7.1 Awareness of the need to protect vulnerable CYP

Few authors went into detail about ethical considerations when involving vulnerable CYP in co‐production.^20^ Authors recognised the need to protect the CYP from harm^37^ through safeguarding and the production of tailored child protection protocols.^18^ The need for more intensive support for vulnerable peer researchers was also highlighted^37^, ^58^ as they frequently have had the same experiences as the interviewees. This creates opportunities for connection between peer researchers and participants but it can also raise difficult emotions if they have had similar traumatic experiences.^38^ It can also lead to peer researchers who are vulnerable CYP becoming more aware of their own experience of oppression or discrimination.^27^

#### 4.7.2 Mechanisms to protect vulnerable CYP

In response to these concerns, in Torronen and Vornanen's study^38^ peer researchers were encouraged to follow the interview topic guide without discussing their own experience, and they did not interview people they knew. Taylor et al.^37^ described how in their study, to support and protect the young peer researchers, they provided debriefing at the end of focus group discussions and team meetings. A known and trusted support worker that they could turn to for support was present in the building for all meetings, training and discussions.

#### 4.7.3 Obtaining ethical approval

The fluid and somewhat unpredictable nature of co‐production with vulnerable CYP can be problematic when trying to navigate systems for obtaining ethical approval, which calls for a detailed and explicit description of all research processes. Liabo et al.^59^ explored the boundaries between protection and participation. While strongly upholding the need for robust ethical approval processes, she argued that these have the potential to limit opportunities for already marginalised CYP to become actively involved in research and have their voices heard. Work with ethics committees and other organisations is needed to enable flexibility of approach while ensuring appropriate safeguarding. This includes making sure that CYP are clear about what they are signing up for and are allowed to discuss with adults, gatekeepers and peers the potential positives and negatives of participation.^30^, ^55^ Having clear goals and protocols so that the expectations of all involved in co‐production are transparent and mutually understood is central to potential participants making an informed decision and not being ‘over‐burdened’.^58^

### 4.8 Areas of best practice in participatory research and co‐production

**Figure 2:**
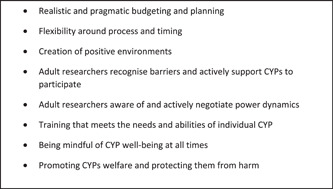
Common principles for participatory research and co‐production. CYP, children and young people.

The co‐production of research with CYP is an evolving area and there are, as yet, no agreed criteria for best practice.^55^ INVOLVE^60^ have published guidance on co‐production which lists five principles: (1) sharing power; (2) including all perspectives and skills; (3) respecting and valuing the knowledge of all when working together; (4) reciprocity and (5) building and maintaining relationships. The findings from this rapid review of co‐produced research with vulnerable CYP strongly echo these five principles. The common principles identified in the articles included in this review are shown in Figure 2.

## 5 DISCUSSION

This review explores the co‐production of health and social science research with vulnerable CYP, the challenges co‐production can present and how these can be overcome. It draws on studies from a wide range of contexts and involves CYP with varied vulnerabilities. What emerges clearly is that co‐production with vulnerable CYP must be led by the key principles of inclusion, safeguarding, respect, well‐being and a real belief in the importance of having CYP's voices heard. To put this into practice, the adult researcher must be willing and able to be reflective and aware of their own biases, expectations, individual strengths and limitations in supporting and facilitating co‐production.

The evidence reviewed demonstrates that supporting vulnerable CYP in the co‐production of research can be challenging, expensive and difficult to organise. For adult researchers, it calls for a flexible way of working and a willingness to relinquish power as an ‘expert’, which may be new and uncomfortable. For CYP, it requires them to give up their time and energy and to expose themselves to situations, dynamics and attitudes that they may not have experienced before. The principles of co‐production identified by INVOLVE^60^ are applicable to all CYP and adults but working with and supporting vulnerable CYP who may have unstable lives, disabilities, health problems, be in care or face other difficulties, presents particular challenges. There may be institutional barriers to overcome and difficult ethical questions posed. How to appropriately protect vulnerable CYP while not restricting their participation in research is an area that needs further consideration.^20^, ^59^

There is a need to develop robust and proportionate ethical approval procedures that facilitate the meaningful participation of vulnerable CYP in research and provide clear guidance on hierarchies of consent for their involvement. This will mitigate against the effective exclusion from participatory research of vulnerable, marginalised CYP. Inclusivity is a core tenet of participatory research but can be difficult to achieve with vulnerable CYP who may need to be reached through institutional and parental gatekeepers. Researchers need to question their own practice to guard against the unnecessary exclusion of young children and those less able to communicate their wants, feelings and experiences.^9^, ^28^

The author S. B. is a member of the study PPI group. She is a care‐experienced young person in the UK who facilitates local authority participation groups for CECYP. She reviewed this report and concluded that it ‘…is pretty accurate when referring to the struggles young people in care and care leavers experience and how that has an effect on their availably and capability to take part in research’. From her experience school, work or education are the main limiters and trying to find enough time after school hours for young people to get involved is difficult.

S. B. confirmed the importance of communication: ‘the more you communicate with care leavers, the more they are willing to listen and be involved’. She advises that researchers need to show understanding, be trauma‐informed and be patient with CECYP. If the young people feel the researcher is not responding to their questions or talking down to them, they will switch off and not want to communicate or participate. Having conversations with those who lead and facilitate participation groups may help researchers prepare for meetings with young people.

She also noted that CECYP have different experiences, capabilities, restrictions and limitations. Co‐production activities and engagement need to be age‐appropriate. It is important for researchers not to make assumptions about CYP capabilities but to decide together the best steps to move forward.

Authors in this review strongly extol the virtues of co‐produced research, pointing to its ability to empower CYP, help them realise their abilities, to positively impact their own communities and wider society^30^, ^35^, ^39^, ^46^ and to become advocates for change.^39^, ^51^ Others point to its ability to transform conventional dynamics between adults and vulnerable CYP.^49^ The benefits of co‐producing research for the individual CYP include acquiring research and life skills^39^, ^43^, ^49^, ^54^ and increased confidence and self‐esteem.^47^, ^54^

Benefits to the research of using a co‐production approach cited include increased relevance,^43^ enabling use of the authentic voice,^55^ making the research more culturally appropriate^30^ and opening adult researchers up to new ideas and perspectives.^38^, ^47^ However, this view is not universal. For example, van Staa et al.,^45^ after weighing up the cost, time and resources required to co‐produce research, expressed doubts about the quality of the research produced by the CYP co‐researchers.

This review has some limitations, which are a reflection of the time‐limited nature of a rapid review. Given the time constraint, methodological choices were made. Only English language peer‐reviewed journals and grey literature were included. Data collection was carried out in an expedited manner by using a single review author with checks by a second review author for data extraction. These choices may have resulted in the omission of relevant data and may have resulted in publication bias. Despite these limitations, the review provides a timely synthesis of the evidence on co‐production of health and social science research with vulnerable CYP, which other researchers will find useful when considering the use of this inclusive approach to research.

There is a lack of established criteria for best practice in using co‐production with CYP. This is an important area of research methodology that warrants further investigation with the goal of reaching a consensus on how best to co‐produce research with these vulnerable CYP.

In this review, a number of papers reflected on the practicalities of co‐producing research with vulnerable CYP. These types of publications are to be encouraged. It is important that those co‐producing research share what they have learnt from the experience ‐ how they have navigated challenges around issues, such as funding and balancing protection and participation, approaches that worked, those that did not and what they would do differently.^12^, ^61^ This needs to be from the perspective not only of adult researchers but also CYP and research managers. In this way, we can learn how we can best meaningfully include vulnerable CYP from diverse backgrounds in research co‐production and decision making.

## 6 CONCLUSION

The co‐production of health and social science with vulnerable CYP can present a number of challenges both for researchers and the CYP themselves. It necessitates the ability to be reflexive and for a flexibility of responsive to unforeseen barriers to engagement. It can test the researchers' preconceptions and demand considerable investment in time and resources. Used appropriately and done well co‐production can offer benefits to all parties involved and contribute to the development of research that reflects the needs of vulnerable CYP.

## AUTHOR CONTRIBUTIONS

**Jo Erwin**: Investigation; writing—original draft; methodology; writing—review and editing; formal analysis; data curation; project administration. **Lorna Burns**: Methodology; writing— review and editing; software; data curation. **Urshla Devalia**: Writing—review and editing. **Robert Witton**: Funding acquisition; writing—review and editing; supervision; conceptualisation. **Jill Shawe**: Funding acquisition; writing—review and editing; conceptualisation. **Hannah Wheat**: Funding acquisition; writing—review and editing; conceptualisation. **Nick Axford**: Writing—review and editing; conceptualisation; funding acquisition. **Janine Doughty**: Funding acquisition; writing—review and editing. **Sarah Kaddour**: Writing—original draft; writing—review and editing. **Abigail Nelder**: Writing—review and editing. **Paul Brocklehurst**: Writing—review and editing. **Skye Boswell**: Writing—review and editing; Validation. **Martha Paisi**: Funding acquisition; writing—review and editing; methodology; conceptualisation; formal analysis; data curation; supervision; project administration.

## CONFLICT OF INTEREST STATEMENT

The authors declare no conflict of interest.

## ETHICS STATEMENT

Ethical approval was not required for this review as data used for analysis was extracted from published studies.

## Supporting information

Supporting information.

Supporting information.

## Data Availability

Data sharing is not applicable to this article as no new data were created or analysed in this study.

## References

[1] Watt RG . Social determinants of oral health inequalities: implications for action. Community Dent Oral Epidemiol. 2012;40(s2):44‐48. 22998304 10.1111/j.1600-0528.2012.00719.x

[2] NICE . Public Health Guidelines on Looked‐After Children and Young People. NICE; 2015.

[3] NHS England . Co‐production. Accessed December 15, 2023. https://www.england.nhs.uk/always-events/co-production

[4] Fieller D , Loughlin M . Stigma, epistemic injustice, and “looked after children”: the need for a new language. J Eval Clin Pract. 2022;28(5):867‐874. 35599388 10.1111/jep.13700 PMC9790323

[5] Public Health England . No Child Left Behind: Understanding and Quantifying Vulnerability. Public Health England; 2020.

[6] Children's Commissioner for England . On Measuring the Number of Vulnerable Children in England. Children's Commissioner for England; 2017.

[7] Bradshaw C , Atkinson S , Doody O . Employing a qualitative description approach in health care research. Glob Qual Nurs Res. 2017;4:233339361774228. 10.1177/2333393617742282 PMC5703087 29204457

[8] Grindell C , Coates E , Croot L , O'Cathain A . The use of co‐production, co‐design and co‐creation to mobilise knowledge in the management of health conditions: a systematic review. BMC Health Serv Res. 2022;22(1):877. 35799251 10.1186/s12913-022-08079-y PMC9264579

[9] Bradbury‐Jones C , Isham L , Taylor J . The complexities and contradictions in participatory research with vulnerable children and young people: a qualitative systematic review. Soc Sci Med. 2018;215:80‐91. 30218806 10.1016/j.socscimed.2018.08.038

[10] Greenhalgh T , Jackson C , Shaw S , Janamian T . Achieving research impact through co‐creation in community‐based health services: literature review and study. Milbank Q. 2016;94(2):392‐429. 27265562 10.1111/1468-0009.12197 PMC4911728

[11] Voorberg WH , Bekkers VJJM , Tummers LG . A systematic review of co‐Creation and co‐production: embarking on the social innovation journey. Public Manag Rev. 2015;17(9):1333‐1357. 10.1080/14719037.2014.930505

[12] Slattery P , Saeri AK , Bragge P . Research co‐design in health: a rapid overview of reviews. Health Res Policy Syst. 2020;18(1):17. 32046728 10.1186/s12961-020-0528-9 PMC7014755

[13] Jull J , Giles A , Graham ID . Community‐based participatory research and integrated knowledge translation: advancing the co‐creation of knowledge. Implement Sci 2017;12(1):150. 29258551 10.1186/s13012-017-0696-3 PMC5735911

[14] Ozer EJ . Youth‐led participatory action research: developmental and equity perspectives. Adv Child Dev Behav. 2016;50:189‐207. 26956074 10.1016/bs.acdb.2015.11.006

[15] Baum F . Participatory action research. J Epidemiol Community Health. 2006;60(10):854‐857. 16973531 10.1136/jech.2004.028662 PMC2566051

[16] Lushey C . Peer Research Methodology: Challenges and Solutions. Sage Publications Inc.; 2017.

[17] van Schelven F , Boeije H , Mariën V , Rademakers J . Patient and Public Involvement of young people with a chronic condition in projects in health and social care: a scoping review. Health Expect. 2020;23(4):789‐801. 32372423 10.1111/hex.13069 PMC7495073

[18] Pavarini G , Lorimer J , Manzini A , Goundrey‐Smith E , Singh I . Co‐producing research with youth: the NeurOx young people's advisory group model. Health Expect. 2019;22:743‐751. 31095837 10.1111/hex.12911 PMC6737761

[19] NICE . Public and Patient Involvement Policy 2023. January 2024. Accessed December 15, 2023. https://www.nice.org.uk/about/nice-communities/nice-and-the-public/public-involvement/public-involvement-programme/patient-public-involvement-policy#principles-for-involving-children-and-young-people

[20] Fløtten KJØ , Guerreiro AIF , Simonelli I , Solevåg AL , Aujoulat I . Adolescent and young adult patients as co‐researchers: a scoping review. Health Expect. 2021;24(4):1044‐1055. 33991369 10.1111/hex.13266 PMC8369088

[21] Afifi RA , Makhoul J , El Hajj T , Nakkash RT . Developing a logic model for youth mental health: participatory research with a refugee community in Beirut. Health Policy Plan. 2011;26:508‐517. 21278370 10.1093/heapol/czr001 PMC3199040

[22] Dadswell A , O'Brien N . Participatory research with care leavers to explore their support experiences during the COVID‐19 pandemic. Brit J Soc Work. 2022;52(6):3639‐3657.

[23] Gray C , Winter E . Hearing voices: participatory research with preschool children with and without disabilities. Eur Early Child Educ Res J. 2011;19(3):309‐320.

[24] Sime D . Ethical and methodological issues in engaging young people living in poverty with participatory research methods. Child Geogr. 2008;6(1):63‐78.

[25] Mawn L , Welsh P , Stain HJ , Windebank P . Youth speak: increasing engagement of young people in mental health research. J Ment Health. 2015;24:271‐275. 26193175 10.3109/09638237.2014.998810

[26] Morris C , Simkiss D , Busk M , et al. Setting research priorities to improve the health of children and young people with neurodisability: a British Academy of Childhood Disability‐James Lind Alliance Research Priority Setting Partnership. BMJ Open. 2015;5:e006233. 10.1136/bmjopen-2014-006233 PMC4316435 25631309

[27] Brady G , Franklin A . Challenging dominant notions of participation and protection through a co‐led disabled young researcher study. J Child Serv. 2019;14(3):174‐185.

[28] Lincoln AK , Borg R , Delman J . Developing a community‐based participatory research model to engage transition age youth using mental health service in research. Fam Community Health. 2015;38(1):87‐97. 25423247 10.1097/FCH.0000000000000054 PMC4256677

[29] Varjavandi R. Blessers must fall: youth‐led participatory action research and photo story creation on teenage pregnancy, transactional sex and gender‐based violence. Agenda. 2017;31(2):87‐98. 10.1080/10130950.2017.1380453

[30] Mitchell K , Durante SE , Pellatt K , Richardson CG , Mathias S , Buxton JA . Naloxone and the Inner City Youth Experience (NICYE): a community‐based participatory research study examining young people's perceptions of the BC take home naloxone program. Harm Reduct J. 2017;14:34. 28592287 10.1186/s12954-017-0160-3 PMC5463299

[31] Ritterbusch AE , Boothby N , Mugumya F , et al. Pushing the limits of child participation in research: reflections from a Youth‐Driven Participatory Action Research (YPAR) Initiative in Uganda. Int J Qual Methods. 2020;19:160940692095896.

[32] Nichols N , Malenfant J. Health system access for precariously housed youth: a participatory youth research project. Soc Ment Health. 2022;12(2):137‐154. 10.1177/21568693221082206

[33] Alderson H , Brown R , Smart D , Lingam R , Dovey‐Pearce G . ‘You've come to children that are in care and given us the opportunity to get our voices heard’: the journey of looked after children and researchers in developing a Patient and Public Involvement group. Health Expect. 2019;22:657‐665. 31115138 10.1111/hex.12904 PMC6737768

[34] Liddiard K , Runswick‐Cole K , Goodley D , Whitney S , Vogelmann E , Watts L . “I was Excited by the Idea of a Project that Focuses on those Unasked Questions” co‐producing disability research with disabled young people. Child Soc. 2019;33(2):154‐167.

[35] Kelly B , Friel S , McShane T , Pinkerton J , Gilligan E. “I haven't read it, I've lived it!”: the benefits and challenges of peer research with young people leaving care. Qual Soc Work. 2020;19(1):108‐124. 10.1177/1473325018800370

[36] Noom MJ , de Winter M , Korf D . The care‐system for homeless youth in The Netherlands: perceptions of youngsters through a peer research approach. Adolescence. 2008;43(170):303‐316. 18689103

[37] Taylor J , Bradbury‐Jones C , Hunter H , Sanford K , Rahilly T , Ibrahim N . Young people's experiences of going missing from care: a qualitative investigation using peer researchers. Child Abuse Review. 2014;23(6):387‐401.

[38] Törrönen ML , Vornanen RH . Young people leaving care: participatory research to improve child welfare practices and the rights of children and young people. Aust Soc Work. 2014;67(1):135‐150.

[39] Garcia AP , Minkler M , Cardenas Z , Grills C , Porter C . Engaging homeless youth in community‐based participatory research: a case study from Skid Row, Los Angeles. Health Promot Pract. 2014;15:18‐27. 23384969 10.1177/1524839912472904

[40] Thulien M , Anderson H , Douglas S , et al. The generative potential of mess in community‐based participatory research with young people who use(d) drugs in Vancouver. Harm Reduct J. 2022;19:30. 35337350 10.1186/s12954-022-00615-7 PMC8956276

[41] Hillier A , Kroehle K . “I'll Save You a Seat”: negotiating power in a Participatory Action Research Project with queer and trans young adults. Urban Educ. 2021;58:004208592110231.

[42] Embleton L , Di Ruggiero E , Odep Okal E , et al. Adapting an evidence‐based gender, livelihoods, and HIV prevention intervention with street‐connected young people in Eldoret, Kenya. Glob Public Health. 2019;14(12):1703‐1717. 10.1080/17441692.2019.1625940 31162989 PMC6906550

[43] Funk A , Van Borek N , Taylor D , Grewal P , Tzemis D , Buxton JA . Climbing the “ladder of participation”: engaging experiential youth in a participatory research project. Can J Public Health. 2012;103(4):e288‐e292. 23618643 10.1007/BF03404237 PMC6973780

[44] Lam GYH , Holden E , Fitzpatrick M , Raffaele Mendez L , Berkman K . “Different but connected”: participatory action research using photovoice to explore well‐being in autistic young adults. Autism. 2020;24:1246‐1259. 31968999 10.1177/1362361319898961

[45] van Staa A , Jedeloo S , Latour JM , Trappenburg MJ . Exciting but exhausting: experiences with participatory research with chronically ill adolescents. Health Expect. 2010;13(1):95‐107. 19682098 10.1111/j.1369-7625.2009.00574.x PMC5060512

[46] Curran T , Jones M , Ferguson S , et al. Disabled young people's hopes and dreams in a rapidly changing society: a co‐production peer research study. Disabil Soc. 2021;36(4):561‐578.

[47] Coser LR , Tozer K , Van Borek N , et al. Finding a voice: participatory research with street‐involved youth in the youth injection prevention project. Health Promot Pract. 2014;15(5):732‐738. 24668583 10.1177/1524839914527294

[48] Kramer J , Barth Y , Curtis K , et al. Involving youth with disabilities in the development and evaluation of a new advocacy training: Project TEAM. Disabil Rehabil. 2013;35(7):614‐622. 10.3109/09638288.2012.705218 22897636

[49] Chappell P , Rule P , Dlamini M , Nkala N . Troubling power dynamics: youth with disabilities as co‐researchers in sexuality research in South Africa. Childhood. 2014;21(3):385‐399.

[50] Burke E , le May A , Kébé F , Flink I , van Reeuwijk M . Experiences of being, and working with, young people with disabilities as peer researchers in Senegal: the impact on data quality, analysis, and well‐being. Qual Social Work. 2018;18(4):583‐600.

[51] Damian AJ , Ponce D , Ortiz‐Siberon A , et al. Understanding the health and Health‐Related social needs of youth experiencing homelessness: a photovoice study. Int J Environ Res Public Health. 2022;19:9799. 36011440 10.3390/ijerph19169799 PMC9408072

[52] INVOLVE . Reward and Recognition for Children and Young People Involved in Research‐Things to Consider. NIHR; 2016.

[53] Nichols J , Klein C , Mwangi D , Kimani S , Braitstein P . Innovative photography‐based approach to evaluating a street children's drop‐in centre from the user's perspective: Kenya. Turk Pediatri Arsivi. 2013;2:30.

[54] Mawn L , Welsh P , Kirkpatrick L , Webster LAD , Stain HJ . Getting it right! Enhancing youth involvement in mental health research. Health Expect. 2016;19(4):908‐919. 26202658 10.1111/hex.12386 PMC5152725

[55] Dovey‐Pearce G , Walker S , Fairgrieve S , Parker M , Rapley T . The burden of proof: the process of involving young people in research. Health Expect. 2019;22(3):465‐474. 30770609 10.1111/hex.12870 PMC6543165

[56] Bailey S , Boddy K , Briscoe S , Morris C . Involving disabled children and young people as partners in research: a systematic review. Dev Med Child Neurol. 2013;2:49. 10.1111/cch.12197 25323964

[57] Nowland R , Robertson L , Farrelly N , et al. Collaborative research methods and best practice with children and young people: protocol for a mixed‐method review of the health and social sciences literature. BMJ Open. 2022;12(10):e061659. 10.1136/bmjopen-2022-061659 PMC9540839 36202582

[58] Dixon J , Ward J , Blower SL . “They sat and actually listened to what we think about the care system”: the use of participation, consultation, peer research and co‐production to raise the voices of young people in and leaving care in England. Child Care Pract. 2019;25(1):6‐21.

[59] Liabo K , Ingold A , Roberts H . Co‐production with “vulnerable” groups: balancing protection and participation. Health Sci Rep. 2018;1(3):e19. 30623060 10.1002/hsr2.19 PMC6266358

[60] Hickey G , Brearley S , Coldham T , et al. Guidance on Co‐producing a Research Project. INVOLVE; 2018.

[61] Amann J , Sleigh J . Too vulnerable to involve? Challenges of engaging vulnerable groups in the co‐production of public services through research. Int J Public Admin. 2021;44(9):715‐727.

