# Supplementary material for: Co‐production of health and social science research with vulnerable children and young people: A rapid review
Source: Health Expect. 2024 Feb 25;27(2):e13991. doi: 10.1111/hex.13991 (PMC10895074; doi:10.1111/hex.13991)
Supplement: Supplementary file 1 — Supporting information. [file HEX-27-e13991-s001.docx]

Supplement 1

Database Search Histories

Date: 27^th^ September 2022

Overview:

| Database | Results |
| --- | --- |
| Embase (Ovid) | **497** |
| MEDLINE (Ovid) | **325** |
| CINAHL (EBSCOhost) | 178  100 (**232** exported from interface) |
| SocINDEX (EBSCOhost) |  |
| Web of Science | **338** |
| Total before deduplication | **1392** |
| Total after deduplication | **813** |

| Embase <1974 to 2022 September 27> | |  |
| --- | --- | --- |
|  |  |  |
| 1 | child*.m_titl. | 955442 |
| 2 | exp *child/ | 151760 |
| 3 | Adolescen*.m_titl. | 239413 |
| 4 | *juvenile/ | 18165 |
| 5 | Teen*.m_titl. | 15395 |
| 6 | (young people or young person* or young adult*).m_titl. | 60479 |
| 7 | youth.m_titl. | 38630 |
| 8 | 1 or 2 or 3 or 4 or 5 or 6 or 7 | 1288580 |
| 9 | vulnerable.tw. | 137407 |
| 10 | vulnerable population/ | 24201 |
| 11 | (transit* adj3 (care or services)).tw. | 16207 |
| 12 | child* in care.tw. | 269 |
| 13 | looked after child*.tw. | 228 |
| 14 | accommodated child.tw. | 2 |
| 15 | ("out of home care" or "out of home placement").tw. | 881 |
| 16 | kinship care.tw. | 184 |
| 17 | ((adoption or adopted) adj3 child*).tw. | 2699 |
| 18 | adopted child/ | 705 |
| 19 | (custod* adj3 care).tw. | 406 |
| 20 | orphan*.tw. | 26066 |
| 21 | (placement adj3 care).tw. | 1376 |
| 22 | public care.tw. | 351 |
| 23 | (foster adj1 (care* or home* or family or parent*)).tw. | 3908 |
| 24 | (institutional* adj3 (care or home*)).tw. | 8332 |
| 25 | (group adj1 home*).tw. | 2129 |
| 26 | (residential adj3 (care or home* or facilit*)).tw. | 10869 |
| 27 | (welfare adj3 (care or system*)).tw. | 3270 |
| 28 | statutory care.tw. | 23 |
| 29 | (care adj3 local authority).tw. | 130 |
| 30 | care order*.tw. | 269 |
| 31 | (substitute adj1 (care or famil*)).tw. | 121 |
| 32 | special guardian*.tw. | 9 |
| 33 | Kafalah.tw. | 1 |
| 34 | (unaccompanied adj3 asylum).tw. | 97 |
| 35 | (unaccompanied adj3 refugee*).tw. | 185 |
| 36 | ("Children Act 1989" or "Children Northern Ireland Order 1995" or "Children Scotland Act 1995").tw. | 57 |
| 37 | (custody or detention).tw. | 8408 |
| 38 | (secure accommodation or secure welfare).tw. | 36 |
| 39 | children in need.tw. | 541 |
| 40 | child protection.tw. | 3136 |
| 41 | special educational need*.tw. | 767 |
| 42 | education health and care plan.tw. | 9 |
| 43 | ((sexual* or physical or emotional*) adj3 abuse*).tw. | 28443 |
| 44 | traffick*.tw. | 80682 |
| 45 | slave*.tw. | 3310 |
| 46 | (victim* adj3 crime*).tw. | 1314 |
| 47 | (FGM or female genital mutilat*).tw. | 2676 |
| 48 | disabilit*.tw. | 316796 |
| 49 | disability/ or physical disability/ | 144310 |
| 50 | ((speech or communication) adj3 (disorder* or difficult*)).tw. | 15719 |
| 51 | (autism or autistic or asperger*).tw. | 78183 |
| 52 | autism/ or exp mental disease/ | 2469893 |
| 53 | physical health.tw. | 35244 |
| 54 | (life limiting or long-term or chronic health or chronic ill*).tw. | 1318526 |
| 55 | exp mental disease/ | 2469893 |
| 56 | (mental health or mental ill* or mental disorder*).tw. | 306808 |
| 57 | CAMHS.tw. | 976 |
| 58 | (homeless* or insecure housing or unstable housing).tw. | 16358 |
| 59 | (free school meals or food poverty or food insecurity).tw. | 7982 |
| 60 | (poverty or impoverished or destitution or deprivation).tw. | 137548 |
| 61 | ((unemploy* adj3 parent*) or low-income or workless).tw. | 50847 |
| 62 | (domestic abuse or domestic violence).tw. | 9104 |
| 63 | (lone parent* or single parent*).tw. | 3565 |
| 64 | (parent* adj capacity).tw. | 242 |
| 65 | ((substance or drug or alcohol) adj (abuse* or misuse*)).tw. | 93797 |
| 66 | (alcoholism or addiction).tw. | 106963 |
| 67 | troubled famil*.tw. | 67 |
| 68 | social work assess*.tw. | 84 |
| 69 | forced marriage*.tw. | 149 |
| 70 | radicalis*.tw. | 381 |
| 71 | ((exclude* or missing) adj2 school*).tw. | 470 |
| 72 | not in education employment or training.tw. | 61 |
| 73 | (gang* or criminal justice or violence* or crime or anti-social or antisocial).tw. | 308115 |
| 74 | (bullying or bullied).tw. | 8998 |
| 75 | (teenage* adj (parent* or mother or mum*)).tw. | 331 |
| 76 | young carer*.tw. | 148 |
| 77 | (LGBT* or Lesbian or Gay or Bisexual or transgender or transsexual).tw. | 31849 |
| 78 | or/9-77 | 4711656 |
| 79 | 8 and 78 | 360319 |
| 80 | patient participation/ and health services research/ | 485 |
| 81 | (participat* adj3 research).ti. | 3318 |
| 82 | participatory research/ | 6678 |
| 83 | (involv* adj3 research).ti. | 1287 |
| 84 | ((co-design* or codesign*) adj3 research).ti. | 17 |
| 85 | (collaborat* adj3 research).ti. | 2429 |
| 86 | ((co-creat* or cocreat*) adj3 research).ti. | 20 |
| 87 | (engag* adj3 research).ti. | 758 |
| 88 | (partner* adj3 research).ti. | 976 |
| 89 | (consult* adj3 research).ti. | 193 |
| 90 | ((co-produc* or coproduc*) adj3 research).ti. | 40 |
| 91 | peer* research*.ti. | 42 |
| 92 | co-research*.ti. | 44 |
| 93 | or/80-92 | 14551 |
| 94 | 79 and 93 | **497** |

| Ovid MEDLINE(R) and Epub Ahead of Print, In-Process, In-Data-Review & Other Non-Indexed Citations, Daily and Versions <1946 to September 26, 2022> | | |
| --- | --- | --- |
|  |  |  |
| 1 | child*.ti. | 836892 |
| 2 | exp *child/ | 3974 |
| 3 | Adolescen*.ti. | 194419 |
| 4 | Teen*.ti. | 12017 |
| 5 | (young people or young person* or young adult*).m_titl. | 47058 |
| 6 | youth.m_titl. | 33056 |
| 7 | or/1-6 | 1053127 |
| 8 | vulnerable.tw. | 107459 |
| 9 | vulnerable population/ | 12580 |
| 10 | (transit* adj3 (care or services)).tw. | 9472 |
| 11 | child* in care.tw. | 208 |
| 12 | looked after child*.tw. | 146 |
| 13 | accommodated child.tw. | 2 |
| 14 | ("out of home care" or "out of home placement").tw. | 812 |
| 15 | kinship care.tw. | 170 |
| 16 | ((adoption or adopted) adj3 child*).tw. | 2259 |
| 17 | adopted child/ | 152 |
| 18 | (custod* adj3 care).tw. | 345 |
| 19 | orphan*.tw. | 19058 |
| 20 | (placement adj3 care).tw. | 1007 |
| 21 | public care.tw. | 289 |
| 22 | (foster adj1 (care* or home* or family or parent*)).tw. | 3455 |
| 23 | (institutional* adj3 (care or home*)).tw. | 5608 |
| 24 | (group adj1 home*).tw. | 1560 |
| 25 | (residential adj3 (care or home* or facilit*)).tw. | 8903 |
| 26 | (welfare adj3 (care or system*)).tw. | 2825 |
| 27 | statutory care.tw. | 19 |
| 28 | (care adj3 local authority).tw. | 105 |
| 29 | care order*.tw. | 134 |
| 30 | (substitute adj1 (care or famil*)).tw. | 112 |
| 31 | special guardian*.tw. | 11 |
| 32 | Kafalah.tw. | 1 |
| 33 | (unaccompanied adj3 asylum).tw. | 61 |
| 34 | (unaccompanied adj3 refugee*).tw. | 162 |
| 35 | ("Children Act 1989" or "Children Northern Ireland Order 1995" or "Children Scotland Act 1995").tw. | 38 |
| 36 | (custody or detention).tw. | 6271 |
| 37 | (secure accommodation or secure welfare).tw. | 19 |
| 38 | children in need.tw. | 432 |
| 39 | child protection.tw. | 2449 |
| 40 | special educational need*.tw. | 437 |
| 41 | education health and care plan.tw. | 4 |
| 42 | ((sexual* or physical or emotional*) adj3 abuse*).tw. | 22183 |
| 43 | traffick*.tw. | 62714 |
| 44 | slave*.tw. | 3193 |
| 45 | (victim* adj3 crime*).tw. | 1056 |
| 46 | (FGM or female genital mutilat*).tw. | 2138 |
| 47 | disabilit*.tw. | 224052 |
| 48 | disabled children/ | 6866 |
| 49 | ((speech or communication) adj3 (disorder* or difficult*)).tw. | 11499 |
| 50 | (autism or autistic or asperger*).tw. | 60897 |
| 51 | exp Neurodevelopmental Disorders/ | 203851 |
| 52 | physical health.tw. | 26913 |
| 53 | (life limiting or long-term or chronic health or chronic ill*).tw. | 959459 |
| 54 | exp mental disorders/ | 1392710 |
| 55 | (mental health or mental ill* or mental disorder*).tw. | 247773 |
| 56 | CAMHS.tw. | 594 |
| 57 | (homeless* or insecure housing or unstable housing).tw. | 13188 |
| 58 | (free school meals or food poverty or food insecurity).tw. | 6543 |
| 59 | (poverty or impoverished or destitution or deprivation).tw. | 109120 |
| 60 | ((unemploy* adj3 parent*) or low-income or workless).tw. | 43418 |
| 61 | (domestic abuse or domestic violence).tw. | 7532 |
| 62 | (lone parent* or single parent*).tw. | 3091 |
| 63 | (parent* adj capacity).tw. | 189 |
| 64 | ((substance or drug or alcohol) adj (abuse* or misuse*)).tw. | 67599 |
| 65 | (alcoholism or addiction).tw. | 76929 |
| 66 | troubled famil*.tw. | 58 |
| 67 | social work assess*.tw. | 48 |
| 68 | forced marriage*.tw. | 138 |
| 69 | radicalis*.tw. | 273 |
| 70 | ((exclude* or missing) adj2 school*).tw. | 288 |
| 71 | not in education employment or training.tw. | 63 |
| 72 | (gang* or criminal justice or violence* or crime or anti-social or antisocial).tw. | 257281 |
| 73 | (bullying or bullied).tw. | 7495 |
| 74 | (teenage* adj (parent* or mother or mum*)).tw. | 302 |
| 75 | young carer*.tw. | 117 |
| 76 | (LGBT* or Lesbian or Gay or Bisexual or transgender or transsexual).tw. | 26873 |
| 77 | or/8-76 | 3198817 |
| 78 | 7 and 77 | 261975 |
| 79 | patient participation/ and health services research/ | 585 |
| 80 | (participat* adj3 research).ti. | 3005 |
| 81 | participatory research/ | 5434 |
| 82 | (involv* adj3 research).ti. | 1173 |
| 83 | ((co-design* or codesign*) adj3 research).ti. | 19 |
| 84 | (collaborat* adj3 research).ti. | 2185 |
| 85 | ((co-creat* or cocreat*) adj3 research).ti. | 25 |
| 86 | (engag* adj3 research).ti. | 703 |
| 87 | (partner* adj3 research).ti. | 868 |
| 88 | (consult* adj3 research).ti. | 168 |
| 89 | ((co-produc* or coproduc*) adj3 research).ti. | 37 |
| 90 | peer* research*.ti. | 27 |
| 91 | co-research*.ti. | 44 |
| 92 | or/79-91 | 12683 |
| 93 | 78 and 92 | **325** |

**CINAHL Plus; (SocINDEX via EBSCOhost)**

| S1 | TI child* or adolescen* or teen* or "young people" or "young person*" or "young adult*" or youth | 641,970 |
| --- | --- | --- |
| S2 | DE "CHILDREN" | 21,014 |
| S3 | S1 OR S2 | 649,306 |
| S4 | TI ( (participat* or involv* or codesign or co-design or collaborat* or cocreat* or co-creat* or engag* or partner* or consult* or coproduc* or co-produc*) N3 research ) OR TI peer* N3 research* OR TI co-research* | 8,865 |
| S5 | S3 AND S4 | 891 |
| S6 | TI (vulnerable OR (transit* N3 (care or services)) or "child* in care" or "looked after child" or "accommodated child*" or "out of home care" or "out of home placement" or "kinship care" or ((adoption or adopted) N3 child*) or custod* N2 care or "kinship care" or orphan* or placement N3 care or "public care" or (foster N1 (care* or home* or family or parent*)) or (institutional* N3 (care or home*)) or group N1 home* or (residential N3 (care or home* or facilit*)) or (welfare N3 (care or system*)) or "statutory care" or (care N3 local authority) or "care order*" or (substitute N1 (care or famil*)) or "special guardian*" or kafalah or (unaccompanied N3 (asylum or refugee*)) or "Children Act 1989" or "Children Northern Ireland Order 1995" or "Children Scotland Act 1995" or custody or detention or "secure accommodation" or "secure welfare" or "children in need" or "child protection" or "special educational need*" or "education health and care plan" or ((sexual* or physical or emotional*) N3 abuse*) OR traffick* or slave* or victim* N3 crime* or FGM or "female genital mutilat*" or disabilit* or disabled or ((speech or communication) N3 (disorder* or difficult*)) or autism or autistic or asperger* or (("life limiting" or "long-term" or chronic or physical or mental) N1 (health or ill* or condition* or disorder*)) OR CAMHS or homeless* or "insecure housing" or "unstable housing" or "free school meals" or "food insecurity" or poverty or impoverished or destitution or deprivation or (unemploy* N3 parent*) or low-income or workless or "domestic abuse" or "domestic violence" or "lone parent*" or "single parent*" or parent* N1 capacity or ((substance or drug or alcohol) N1 (abuse* or misuse*)) or alcoholism or addiction or "troubled famil*" or "social work assess*" or "forced marriage*" or radicalis* or ((exclude* or missing) N2 school*) or "not in education employment or training" or gang* or "criminal justice" or violence* or crime or anti-social or antisocial or bullying or bullied or (teenage* N1 (parent* or mother or mum*)) or "young carer*" or LGBT* or Lesbian or Gay or Bisexual or transgender or transsexual) | 480,483 |
| S7 | AB (vulnerable OR (transit* N3 (care or services)) or "child* in care" or "looked after child" or "accommodated child*" or "out of home care" or "out of home placement" or "kinship care" or ((adoption or adopted) N3 child*) or custod* N2 care or "kinship care" or orphan* or placement N3 care or "public care" or (foster N1 (care* or home* or family or parent*)) or (institutional* N3 (care or home*)) or group N1 home* or (residential N3 (care or home* or facilit*)) or (welfare N3 (care or system*)) or "statutory care" or (care N3 local authority) or "care order*" or (substitute N1 (care or famil*)) or "special guardian*" or kafalah or (unaccompanied N3 (asylum or refugee*)) or "Children Act 1989" or "Children Northern Ireland Order 1995" or "Children Scotland Act 1995" or custody or detention or "secure accommodation" or "secure welfare" or "children in need" or "child protection" or "special educational need*" or "education health and care plan" or ((sexual* or physical or emotional*) N3 abuse*) OR traffick* or slave* or victim* N3 crime* or FGM or "female genital mutilat*" or disabilit* or disabled or ((speech or communication) N3 (disorder* or difficult*)) or autism or autistic or asperger* or (("life limiting" or "long-term" or chronic or physical or mental) N1 (health or ill* or condition* or disorder*)) OR CAMHS or homeless* or "insecure housing" or "unstable housing" or "free school meals" or "food insecurity" or poverty or impoverished or destitution or deprivation or (unemploy* N3 parent*) or low-income or workless or "domestic abuse" or "domestic violence" or "lone parent*" or "single parent*" or parent* N1 capacity or ((substance or drug or alcohol) N1 (abuse* or misuse*)) or alcoholism or addiction or "troubled famil*" or "social work assess*" or "forced marriage*" or radicalis* or ((exclude* or missing) N2 school*) or "not in education employment or training" or gang* or "criminal justice" or violence* or crime or anti-social or antisocial or bullying or bullied or (teenage* N1 (parent* or mother or mum*)) or "young carer*" or LGBT* or Lesbian or Gay or Bisexual or transgender or transsexual) | 939,870 |
| S8 | S6 OR S7 | 1,089,397 |
| S9 | S3 AND S4 AND S8 | **278** |

**Web of Science**

| TI=(child* or adolescen* or teen* or "young people" or "young person*" or "young adult*" or youth) |
| --- |
| TI=((participat* or involv* or codesign or co-design or collaborat* or cocreat* or co-creat* or engag* or partner* or consult* or coproduc* or co-produc*) near/3 research)) |
| TS=( (vulnerable OR (transit* NEAR/3 (care or services)) or "child* in care" or "looked after child" or "accommodated child*" or "out of home care" or "out of home placement" or "kinship care" or ((adoption or adopted) NEAR/3 child*) or custod* NEAR/2 care or "kinship care" or orphan* or placement NEAR/3 care or "public care" or (foster NEAR/1 (care* or home* or family or parent*)) or (institutional* NEAR/3 (care or home*)) or group NEAR/1 home* or (residential NEAR/3 (care or home* or facilit*)) or (welfare NEAR/3 (care or system*)) or "statutory care" or (care NEAR/3 local authority) or "care order*" or (substitute NEAR/1 (care or famil*)) or "special guardian*" or kafala or (unaccompanied NEAR/3 (asylum or refugee*)) or "Children Act 1989" or "Children Northern Ireland Order 1995" or "Children Scotland Act 1995" or custody or detention or "secure accommodation" or "secure welfare" or "children in need" or "child protection" or "special educational need*" or "education health and care plan" or ((sexual* or physical or emotional*) NEAR/3 abuse*) OR traffick* or slave* or victim* NEAR/3 crime* or FGM or "female genital mutilat*" or disabilit* or disabled or ((speech or communication) NEAR/3 (disorder* or difficult*)) or autism or autistic or asperger* or (("life limiting" or "long-term" or chronic or physical or mental) NEAR/1 (health or ill* or condition* or disorder*)) OR CAMHS or homeless* or "insecure housing" or "unstable housing" or "free school meals" or "food insecurity" or poverty or impoverished or destitution or deprivation or (unemploy* NEAR/3 parent*) or low-income or wordless or "domestic abuse" or "domestic violence" or "lone parent*" or "single parent*" or parent* NEAR/1 capacity or ((substance or drug or alcohol) NEAR/1 (abuse* or misuse*)) or alcoholism or addiction or "troubled famil*" or "social work assess*" or "forced marriage*" or radicalis* or ((exclude* or missing) NEAR/2 school*) or "not in education employment or training" or gang* or "criminal justice" or violence* or crime or anti-social or antisocial or bullying or bullied or (teenage* NEAR/1 (parent* or mother or mum*)) or "young carer*" or LGBT* or Lesbian or Gay or Bisexual or transgender or transsexual))  **338** |
