# Supplementary material for: Co‐production of health and social science research with vulnerable children and young people: A rapid review
Source: Health Expect. 2024 Feb 25;27(2):e13991. doi: 10.1111/hex.13991 (PMC10895074; doi:10.1111/hex.13991)
Supplement: Supplementary file 2 — Supporting information. [file HEX-27-e13991-s002.docx]

Supplement 2:

Study details

| **Author(s)** | **Year** | **Aims/objectives** | **Document type** | **Study design** | **Country** | **Vulnerability** | Age | **No. actively involved in co-production** | **Co-production approach** | **Activities** | **Challenges to co-production** | **How challenges overcome** |
| --- | --- | --- | --- | --- | --- | --- | --- | --- | --- | --- | --- | --- |
| Afifi, R. A., Makhoul, J. ,El Hajj, T., Nakkash, R. T. | 2011 | To develop a logic model for a youth mental health promotion intervention using a participatory approach in a Palestinian refugee camp in Beirut, Lebanon. | Descriptive | Qualitative | Lebanon | Refugee | 17-25 | 2 | Participatory | A Community Youth Committee (CYC) was formed. Participation of youth on the CYC was perceived by all members to be a priority. Therefore 18 young men and women aged 17–25 years—identified by the participating NGOs as active in their community—were invited to attend the CYC meetings. Five attended regularly but were not active participants, due to the cultural deference to age. In order to empower the youth, a Palestinian Youth Coalition (PYC) was established in Burj El Barajneh camp. This coalition included all the youth (18) and selected two to represent them at CYC meetings. The CYC prioritised health issues, explored determinants, agreed on criteria for selecting intervention, selected intervention to adapt to context, culturally adapted activities and developed intervention package. | In communities that are patriarchal, cultural norms may prevent youth from speaking vocally in front of adults, especially when they disagree. | The youth created their own committee where they felt free to talk and empowered two representatives to attend the CYC meetings. |
| Alderson, H., Brown, R., Smart, D., Lingam, R., Dovey-Pearce, G. | 2019 | Looked after children and care leavers (denoted as LAC) are often described as a ‘hard to reach’ group of young people, and their voices are rarely sought to inform academic research. This paper reports on experiences and reflections of a group of children and young people and academic researchers who developed a Patient and Public Involvement (PPI) group that was set up in the context of an ongoing health service intervention trial with LAC. | Reflective | Qualitative | UK | Looked after children | 15-19 | 11 | PPI | The LAC used the PPI group to produce a 5‐minute video to highlight why they think young people should be involved in research. Findings from the research were used to co‐develop ‘top tips’ of working with vulnerable young people such as looked after children. Series of discussion meetings held at (but separate to) the routine CICC meetings | Researchers "need resilience, patience and tolerance" to work with LAC. | Important that participants can understand how their input has influenced the project. Important was the opportunity to engage with an existing group where LAC were represented and having a familiar face (a participation officer) to help overcome some of the barriers to engagement. |
| Bailey, S., Boddy, K., Briscoe, S., McHugh, C., Stone, T., East, A., Morris, C. | 2014 | To carry out a systematic review of how to Involve disabled children and young people as partners in research | Review | Systematic review | World | Disabled | 5-25 | N/A | Participatory research | N/A | Communication –  Anticipating and meeting the communication and access needs of CYP; DCYP need effective ways of communicating with each other, and with research staff . A lack of suitable aids can result in those who use nonverbal communication being excluded.  Experience & balance of power - Researchers need to balance the CYP’s right to participate with their responsibility to protect them and ensure they are not overburdened. Sensitive meeting content may cause distress, it may not be possible to guarantee confidentiality in project meetings. A lack of confidence feeling intimidated by professionals or unfamiliar working environments, unrealistic expectations of involvement, feelings of tokenism.  Gatekeepers - may discourage, prevent or forget about involvement, speak on the CYP’s behalf | Many challenges can be overcome  with sufficient time, planning and resources.  Communication – Use of Drawing, photography, talking mats, cue cards, pictures and tape recordings to engage non-verbal CYP. A trusted adult or familiar communicator can be useful to facilitate and support communication. Research staff require sufficient time, resources, skills and training to facilitate successful involvement.  Experience & balance of power – empower CYP  to take control over the research agenda and methods, define their own and others’ roles in the project. Allow them to raise issues that are important to them. Be transparent if a certain option is not possible. Give feedback.  Gatekeepers - should be fully informed of their roles and  Responsibilities & encouraged to act as advocates for their CYP’s involvement |
| Bradbury-Jones, C., Isham, L.,Taylor, J. | 2017 | A qualitative systematic literature review exploring core ethical and methodological issues involved in carrying out participatory research with vulnerable children and young people. | Review | Systematic review | World | Vulnerable CYP | NI | N/A | Participatory research | N/A | Inclusion: older and more articulate children are more likely to participate than those who are young and less articulate. Also, children with more complex social and communications needs are less visible in participatory research also children from minority ethnic groups.  Over-researched –over-inclusion of certain groups | Inclusion - Researchers to think creatively in terms of how to engage with children and young people at the margins.  Power – “to view the vulnerable children and young people as powerful agents, capable of exerting political influence” |
| Brady, G., Franklin, A. | 2019 | To present a research process designed to address issues around the inclusion of disabled children and young people in decisions about their own support and within research processes. Training and supporting a group of disabled young people to co-lead, undertake and share research which defines quality and rights-based Education, Health and Care Plans (EHCPs); and supporting disabled young people to develop a framework and resources to ensure that quality and rights are at the forefront in the development and review of EHCPs. | Descriptive | Qualitative | UK | Disabled | 16-23 | 6 | Peer research | Defined research agenda, undertook field work, co-designed and co-produced all project outputs and resources. | Tension between duty of care and autonomy - changed the dynamics. | Where possible communicated only with YP to respect their autonomy |
| Burke, E., le May, A., Kébé, F., Flink, I., van Reeuwijk, M. | 2018 | To explore how the peer approach in a study of access to sexual and reproductive health services for young people with disabilities (YPWD) affected the quality of data collected and data analysis and the experiences of the peer researchers throughout the process by using reflexivity tools. | Reflective | Qualitative | Senegal | Physical disability | NI | 14 people were trained: 3 were visually impaired, 9 had a physical impairment and 2 were sign language interpreters. | Peer research | Peer researchers were responsible for transcribing FGDs and interviews, thematic analysis. Peer researchers also had the opportunity to present conclusions of their preliminary research findings. | Some peer researchers struggled and lacked confidence in data analysis. Not all peer researchers were equally capable to analyse data, insufficient time and support was allocated to this activity. During training peer researchers struggled to draw clear conclusions from their practice FGD and interview findings. | Given the time available, during the analysis of field data more focus was placed on the presentation of results as opposed to recommendations. |
| Chappell, P., Rule, P., Dlamini, M., Nkala, N. | 2014 | To understand what youth with disabilities learn through undertaking sexuality research as co-researchers | Reflective | Qualitative | South Africa | Physical disability | 15 to 20 | 3 | Co-production | Carried out single-sex and mixed-sex focus group discussions and individual interviews with other youth with disabilities, as well as being involved in some aspects of the data analysis of the study. |  |  |
| Coser, L. R., Tozer, K., Van Borek, N., Tzemis, D., Taylor, D. et al. | 2014 | This article uses a Positive Youth Development framework to explore the experiences of six experiential youth coresearchers (YCs) in the Youth Injection Prevention (YIP) participatory research project, and the parallel track process of empowerment and capacity building that developed. | Reflective | Qualitative | Canada | Street involved youth | 17 to 24 | 6 | Participatory | Facilitating focus groups with street-involved youth, note taking, assisting with open coding exercises with interview transcripts, identifying and discussing key themes with the project team, identifying the most pertinent and representative quotes for inclusion in presentations, hosting fund-raising events, and disseminating results at scientific conferences and community forums | Time required to help YCs overcome personal barriers to participation. For most youth, was first time had worked in a participatory environment. The process of group engagement was a challenge, particularly in forming consensus and hearing everyone’s opinions. Being inclusive and reaching consensus required them to be less judgemental and to develop listening skills and patience. YCs participation often mediated through difficulties in their personal lives. The provisions made to accommodate and support them in difficult times affected project timelines and budget. | Team-building activities and regular meetings were key to maintaining group cohesion. Engagement strengthened by providing a supportive environment, food, coffee, and frequent breaks throughout meetings. Needed to give YCs more than basic skills training; encouragement and support in both the research process and their personal lives were essential for maintaining the YCs’ participation. Pace and content of training needs to reflect the different abilities and needs of the participants. |
| Curran, T., Jones, M., Ferguson, S., Reed, M., Lawrence, A., et al. | 2021 | To support disabled young people to explore and re-imagine their transitions into adulthood. | Descriptive | Qualitative | UK | Disability | NI | 11 | Peer research | The research group ran knowledge café events through a college and youth club with 57 disabled young people. Members of the research group made notes and took photographs during the knowledge café events. Attended reflection meetings to start identifying themes from the fieldwork. Developed key messages. |  |  |
| Dadswell, A., O’Brien, N. | 2022 | To explore care leavers’ support experiences during the COVID-19 pandemic. | Descriptive | Qualitative | UK | Care leavers | 16 to 25 | 25 | Participatory | Identified priorities and developed an online questionnaire, participated in focus groups, reflected on findings, agreed recommendations and developed resources from the research. Participated in a conference for the wider project, including leading breakout room discussions. | NI | NI |
| Damian, A. J., Ponce, D., Ortiz-Siberon, A., Kokan, Z., Curran, R., Azevedo, B., Gonzalez, M. | 2022 | To obtain a nuanced understanding of the individual, familial, community and societal factors that contribute to the health and health-related needs of youths experiencing homelessness. | Descriptive | Qualitative | USA | Homeless | 14 -24 | 14 | Co-production | (1) photovoice trainings, (2) phototaking fieldwork, (3) one-on-one meetings, and (4) group sharing and analysis | Reasons for not participating in the training sessions included conflicts with their work schedule, disruptions resulting from the experience of homelessness, such as not having a space to attend sessions with WIFI access, and family commitments | Thorough and rigorous training meant that youth researchers were capable of conducting effective interviews |
| Dixon, J., Ward, J., Blower, S. | 2019 | This paper discusses the development of methodologies for hearing and acting upon the voices of care-experienced children and young people. It describes four different approaches; participation, consultation, peer research and co-production and present examples from their own research. | Reflective | N/A | UK | Young people in care and care leavers | N/A | N/A | N/A | N/A | Ethical and fieldwork safety issues, highlighting the need for more intensive support for care experienced peer researchers. Many young people will have limited experience of research. Cost and need for flexibility. | Training and support for peer researchers, to include strategies for conducting research interviews safely for researchers and participants. Practicing strategies for safely ending interviews that might become upsetting. Provide tailored training and support.  Open and honest communication. Having realistic timescales, costs and flexibility. |
| Dovey-Pearce, G., Walker, S., Fairgrieve, S., Parker, M., Rapley, T. | 2018 | This study describes the experiences of adult researchers and young people involved in a large- scale, UK health research programme (the "Transition" study) exploring the process of working together and the outcomes of that work. The “Transition” study was a 5- year longitudinal health research programme, , examining how health services in the UK can support young people in their move from childhood to adulthood. It comprised nine work packages, with one focusing on young people’s involvement in the programme. | Reflective | Mixed methods | UK | Experience of living with physical and/or developmental conditions | 15-20 | 20 | PPI | Most meetings had approx 8 attendees. The UP Group’s role was to provide a young people’s perspective, with the aim of working with the adult researchers to oversee the governance and delivery of the Transition programme. The involvement lead was a member of the research team and reported to the research management meetings with the young people attending these meetings, when adults or young people felt there was a need. | For the adult researchers, sometimes understanding how best to involve the young people was anxiety inducing. Power imbalance. Managers wanted work done at pace, felt were slowed down by the young peoples involvement. The young people had doubts about meeting new people and being in a group, not used to formal meetings. | Members of the management group invited to some of early sessions to help them understand nature of interaction Young people preferred face-to-face meetings. YP had a work stream that they led on and were supported in this work by the involvement facilitators with little direct input from the research team. All aspects of the YP’s work were outlined at the formal research management meetings, initially by the involvement lead and then with YP attending themselves. |
| Embleton, L., Di Ruggiero, E., Odep Okal, E., Chan, A. K., Logie, C. H., , Ayuku, D., Braitstein, P. | 2019 | To describe the participatory research process of adapting the evidence-based combined Stepping Stones and Creating Future interventions with street connected young people SCY in a new setting in Eldoret, Kenya using a modified ADAPT-ITT model. | Reflective | Mixed-methods | Kenya | Street-connected, i.e. spent time on street in past 6 months and not enrolled or attending school | 16-24 | 24 | Participatory research | Took part in FGD which were facilitated by peer facilitators who were also SCY. Peer facilitators and other SCY contributed to decisions re adaptation of the programmes and contributed to intervention development and identifying health priorities, also illustrated the intervention manual. | NI | NI |
| Fløtten, KjØ., Guerreiro, A. I. F., Simonelli, I., Solevåg, A. L., Aujoulat, I. | 2021 | To summarize empirical evidence and identify knowledge gaps about the involvement of young patients (adolescents and young adults) as co- researchers. | Review | Scoping review | World | CYP who are patients | Aged 12+ | N/A | Participatory | N/A | Need for time, funding and flexibility when including young patients as co-researchers. Knowledge gaps concern legal and ethical dilemmas of including a vulnerable group as co-researchers. | More reflection is needed about what meaningful participation is and what it entails in this context.  Safeguarding of co-researcher - training; self- care plan so that possible challenges are discussed and addressed ahead of time; flexibility. |
| Funk, A. Van Borek, N., Taylor, D., Grewal, P., Tzemis, D., Buxton, J. | 2013 | The Youth Injection Prevention (YIP) Project sought to identify factors that prevented street-involved youth from moving into injection drug use in Vancouver, BC; paper describes the level of participation observed among the street-involved youth taking part in our project. | Reflective | Qualitative | Canada | Street involved youth | 19-24 | 6 | Participatory | The youth co-researchers provided input into the interview and focus group guides for face validity. Also carried out analysis (coding). 4 YP presented at national public health conference. | Challenges include time limitations and steep learning curves. To accommodate increasing participation and differential learning during the project, extra sessions were created and deadlines extended, which had budget implications. Despite this it was not feasible to fully involve the youth in all aspects of the YIP project, as many methodological techniques were too advanced to teach in short time frame and not all youth were interested in or capable of long work hours. | Flexibility of the project and mutual understanding between the youth and project coordinators, which was strengthened through training and team-building sessions. Not all youth participated at the same level at each stage of the project. Flexibility in movement between levels of participation allowed youth to determine their own level of involvement based on their ability. |
| Garcia, A., Minkler, M., Cardenas, Z., Grills, C., Porter, C. | 2014 | To examine a youth-focused CBPR project where a core group of 15 African American and Latino young people worked with adult mentors at a local community-based organization and a university to study and address the concerns of youth in their neighborhood | Reflective | Qualitative | USA | Homeless | 11 to 19 | 15 | CBPR | Design of and administering survey; interpretation of data. | Need enough resources to maintain momentum of the group; Distrust of adults who have failed them. | Working successfully with young people requires a special kind of person who respects youth culture and is committed to bringing out young people's abilities. Adult mentors who were able to break through these barriers by establishing relationships through culturally grounded interventions, mutual trust, respect, and communication |
| Garcia-Quiroga, M., Agoglia, I. | 2020 | To review the experience of researchers and evidence re participation in research by children in alternative care and adoption | Reflective | Qualitative |  |  |  |  |  |  |  |  |
| Gray, C., Winter, E. | 2011 | To involve children with and without a disability in every stage of the research; to determine children’s understanding, time on task and engagement in the research process; to explore within and between group interactions to determine children’s level ofengagement and ownership of the research. | Descriptive | Qualitative | Northern Ireland | Disability | 3 to 4 years | 36 (18 disabled and 18 non-disabled) | Participatory | Chose issue of importance to their lives and selected research tools, collected data and disseminated findings. | The open time frame adopted was detrimental to children with attention disorders. They quickly became tired and frustrated and exhibited behaviours that could impact negatively upon their peers. | Imposed a time frame |
| Hillier, A., Kroehle, K. | 2021 | To describe the research process in an empirical research project with queer and trans youth of colour about the experiences of trans students with urban public high schools. | Reflective | Qualitative | USA | Queer & trans | NI | 4 | YPAR | Participated in reviewing and testing the semi-structured interview guide. The final guide included questions about relationships with classmates, faculty, family, and school staff, travel to and from school, curricula, extracurricular activities, school facilities, administrative documents, and gender-affirming resources. Each interview was led by one of two youth interns. Our team also produced a 9-minute video3 describing the creation of Policy 252 and our hopes for full implementation. The youth proposed the video project, did most of the filming and editing, and narrated the video. | YPAR collaboration pushed up against academic conventions about who should serve as co-authors and what a co-authorship process entails. Involved more time and negotiation around who makes revisions, how they are made, and where (physically) collaboration takes place; Conflict between formal credentials and lived experience. | Challenged adultism by centering—and compensating—youth expertise as co-authors, interviewers, artists, and video producers. Used collaborative decision making and knowledge production. |
| Kelly, B., Friel, S., McShane, T., Pinkerton, J., Gilligan, E. | 2018 | This article aims to add to that understanding by providing a detailed account and reflection of the involvement of care leavers as peer researchers in the qualitative case study phase of a three-year, mixed method study of the transitions of young people leaving care in Northern Ireland | Reflective | Qualitative | UK | LAC | Early 20s | 4 (6 dropped out) | Peer research | Interviewing peers, co-produced interview schedules | Some aspects of the interview that peer researchers found more challenging, such as asking about mental health/disability issues or unfamiliar services. | The peer researchers needed to build their confidence to say when they did not know about a service and ask the participant to provide further information |
| Kramer, J., Barth, Y., Curtis, K., Livingston, K., O'Neil, M. et al. | 2013 | To describe a participatory research process in which six youth with disabilities (Youth Panel) participated in the development and evaluation of a manualized advocacy training, Project TEAM (Teens making Environment and Activity Modifications). | Reflective | Intervention | USA | Physical disability , Autism, ADHD | 12 to 16 | 6 | Participatory | The Youth Panel designed and administered a survey and focus group to evaluate enjoyment and usefulness of Project TEAM with support from an advocate/researcher. Members of the Youth Panel analysed survey response frequencies. | NI | NI |
| Lam, G. Y. H., Holden, E.,Fitzpatrick, M.,Raffaele Mendez, L.,Berkman, K. | 2020 | To use Photovoice to engage autistic young adults to express and communicate their ideas about well-being. | Descriptive | Qualitative | USA | Autism | 19-25 | 14 | PAR | Taking photos, making research decisions, analysing data. | The degree to which individuals were willing to be involved in research activities varied. | NI |
| Larkins, C., Nowland, R., Robertson, L., Nicola Farrelly, Sharpe, D., Roy, A. et al. | 2021 | To carry out a rapid literature review of Peer Research by Children and Young  People and their allies | Review | Rapid review | World | All CYP including vulnerable CYP | 5-25 | N/A | Peer research | N/A | Time; power relations; negotiating commitment and challenges; managing collaboration & capacity; credibility & achieving change. | Projects must challenge the normative conventions of  research as well as traditional methods and methodologies. Adults who take part must be  open minded, flexible, easy-going, compassionate and willing to be equal partners. They must balance strike a balance with protection and enabling inclusion  and must co-develop strategies which support young people throughout the different stages  of projects. |
| Laws S., Mann G. | 2004 | To encourage meaningful and ethical participation by children in research related to violence against children. | Toolkit | N/A | World | CYP | NI | N/A | Participatory | N/A | Inclusivity; mamnaging expectations; safeguarding. | Consider the risks and costs to children of their participation. ie: time, inconvenience, embarrassment, sense of failure or coercion,  fear of admitting anxiety, pressure to perform unachievable tasks. Act always  in their best interests.  Build in benefits for children who choose to become involved in  research.  •Explore with children what you and they think are appropriate roles for adults  and children in the work. Share goals and expectations with each other. Draw up a group contract or ground  rules to set the stage for your work together. |
| Liabo, K., Ingold, A., Roberts, H. | 2018 | To explore the tension between participation and protection at a time when professionals are encouraged to engage patients and citizens in both the “R” (research) and the “D” (development) of services. | Reflective | Qualitative | UK | Care leavers | 16-24 | 24 | Participatory | Data collection through participatory meetings | The risk reduction strategies that often underpin ethics approval processes can  carry the risk of limiting opportunities to play a part in research for people who may already be  excluded on age, health, language, or other grounds. It risks reducing the range of lay knowledge on which  researchers can draw, limits generalisability, and potentially adds to damaging social exclusion. | Enabling CYP to participate effectively as a life skill. Ethics committees realising the role of CYP as active citizens. Ethics committees and researchers need to promote and conduct inclusive  research that responds to participants' needs to have their participation well supported. |
| Liddiard, K., Runswick-Cole, K., Goodley, D., Whitney, S., Vogelmann, E., Watts, L. | 2018 | To detail the politics and practicalities of co-produced disability research with disabled young people with life-limiting and life-threatening impairments, centering on an arts informed co-produced research project that has brought together a Co-Researcher Collective of disabled young people. | Reflective | Qualitative | UK | Life limiting or life threatening impairments |  | 6 | Co-production | (i) supporting research design (ii) co-writing interview schedules; (iii) recruiting participants for data collection and carrying out interviews (iv) planning the project’s impact strategy and building relationships with impact partner organisations; (v) working with community research partner organisations; (vi) meeting with Research Management Team to co-manage the research process as a whole; (vii) writing blogs, making films to communicate processes and preliminary findings; (viii) presenting at conferences and research festivals; (ix) undertaking public engagement and knowledge translation activities; (x) co-authoring articles for publication. | YP have busy lives which more important to them than research. Having session within school (and school time) likely emphasised the project as another form of schoolwork. The students who came to the session were all post-16 and thus busy with the current demands of GCSEs, A-Levels and BTEC learning. | Used online advertising through the project website and social media which led to prospective Co-Researchers making initial contact |
| Lincoln, A., Borg, R., Delman, J. | 2015 | To present a model for the development and conduct of a Community-Based Participatory Research (CBPR) project with transition age youth (TAY) mental health service users. | Descriptive | Qualitative | USA | Mental health | 18-25 | 6 | CBPR | Literature review, defining problem, development of interview guide, development of criteria for eligibility, recruitment, data collection, data analysis, dissemination | Disclosure – Community Research Assistants (CRBs) felt varying levels of discomfort about disclosing themselves as mental health service users. Institutional review board (IRB) lack of understanding of mental health and how it may impact training of CRAs. Interviews may elicit difficult topics which may be painful for CRAs | Partnered and worked with IRB to discuss these issues. Have self-care plans for CRAs in place. |
| Mawn, L., Welsh, P., Stain, H. J., Windebank, P. | 2015 | The development of a youth research group (Youth Speak) aimed at increasing youth engagement in mental health research | Reflective | Various | UK | Experienced mental health problems or are carer/sibling of someone with mental health problems or no personal experience of mental health issues. |  | 20, approx 6-8 attend each meeting. | PPI | Members of Youth Speak meet on a monthly basis to discuss research priorities, feedback information from local and national conferences, and collaborate with academics and clinicians. All activities are co-ordinated by academic (adult) researchers with contact between meetings occurring via email and social media. | Lack of specific funding initiatives targeting enhanced and sustained PPI; Delivering PPI that develops a youth culture can be expensive and have work to work around YPs complex lives. Young people come and go from the group resulting in fluctuating numbers and membership. Identifying young people who want to be involved in research can be time consuming and difficult. Poor involvement of young people can lead to a loss of self-confidence and a negative view of research. Research involving young people can be difficult to navigate through research ethics committees. | In promoting a youth culture, meetings are held outside of school and college hours in public buildings (town halls or university buildings) that are non-stigmatising and are highly accessible by public transport. At each meeting we provide food, refreshments and icebreaker activities, while the young members set the agenda, determine priorities and run the meeting. Minimising disruption caused by YP leaving the project is key to the success of the group, thus on-going recruitment is necessary. |
| Mitchell, K., Durante, S. E., Pellatt, K., Richardson, C. G., , Mathias, S., Buxton, J. A. | 2017 | To explore how street-involved young people experience the THN Program in Vancouver Canada | Descriptive | Qualitative | Canada | Street-connected | NI | 2 | CBPR | Research design, interviewing, data interpretation, dissemination including contributing to video. | NI | NI |
| Morris, C., Simkiss, D., Busk, M., ,Morris, M., Allard, A., Denness, J., Janssens, A. et al. | 2014 | To engage young people, parent carers and clinicians in a systematic process to identify and prioritise research questions regarding ways to improve the health and well-being of children and young people with neurodisability. | Descriptive | N/A | UK | Neurodisability | NI | 4 | PPI | Participation in steering group and workshop | NI | NI |
| Nichols, N., Malenfant, J. | 2022 | Our participatory youth research team explored homeless youth’s health-seeking practices, the specific barriers they face, and relations between their health-seeking efforts and their homelessness. | Descriptive | Qualitative | Canada | Homelessness experienced youth | NI | 4 | Participatory | Research co-design, Interviewing | NI | Team building. Spent time each week sharing experiences and mobilizing expertise within the group. |
| Noom, M. J., de Winter, M., Korf, D. | 2008 | To examine the perceptions of homeless youth of the care they receive. | Descriptive | Qualitative | Netherlands | Homeless | 15 to 24 years | 22 | Peer research | Interviewing peers | NI | NI |
| Pavarini, G., Lorimer, J., Manzini, A., Goundrey-Smith, E., Singh, I. | 2019 | To provide a step-by-step model, grounded in our own experience of setting up and coordinating the Oxford Neuroscience, Ethics and Society Young People's Advisory Group (NeurOx YPAG). This group supports studies at the intersection of ethics, mental health and novel technologies. Our model covers the following stages: deciding on the fit for co-production, recruiting participants, developing collective principles of work, running a meeting and evaluating impact | Reflective | Various | UK | Mental health | NI | NI | Co-production | Meetings to discuss research design and other activities e.g. presentation, contribution to articles. | Some young people work or have other extra curricular activities so constrained as to how much can take part. | Flexible scheduling. Offer additional support to those who might not have some of the skills needed for participation, different roles for different strengths. |
| Ritterbusch, A. E., Boothby, N., Mugumya, F., Wanican, J., Bangirana, C. et.al. | 2020 | Part of large scale qualitative study on violence against childre ocurring in and outside of households. Aim to utilize engagement with YPAR to reflect on methodological best practices for violence against children research that involve children themselves as part of a movement to democratize the research process. | Descriptive | Qualitative | Uganda | Street connected | 16 to 25 | 4 | YPAR | Youth researchers (YR) recruited partcipants, selected methods of data collection, designed data collection tools, collected, analysed and disseminated data | Sustainability - cost of this approach | Include money in budget for YR's salary, invest in their long term professional development, showcase their achievements. |
| Sime, D. | 2008 | To discuss the methodological and ethical issues arising from a project that focused on conducting a qualitative study using participatory techniques with children and young people living in disadvantage. The main aim of the study was to explore the impact of poverty on children and young people’s access to public and private services. | Reflective | Qualitative | UK | Low income | 10 to 14 | 10 | YPAG | Prior to writing the proposal, a group young people were involved in a planning meeting, with the aim of informing the research on the key aspects related to young people’s awareness of and use of services and to identify issues that young people themselves deemed as important in relation to service provision and service use in deprived areas. Children’s involvement in decisions about the main aspects of the research and its methodological design was key to the project. | Negotiating access at multiple levels with the adults that control the spaces suitable to conduct research with children, such as schools or children’s clubs. | Depended on researcher’s personal skills and ability to convince the gatekeeper, usually in short phone conversations, that the research was worthwhile doing. Used verbal rather than written consent when speaking to mothers on the phone. |
| Taylor, Julie Bradbury-Jones, C., Hunter, H., Sanford, K., Rahilly, T., Ibrahim, N. | 2014 | To investigate young people's experiences of going missing from care and to identify the issues that contributed to them running away; trigger factors that prompted episodes of going missing; support received during or following instances where they went missing; and factors that might prevent future absconding. | Descriptive | Qualitative | UK | Children in care | Ages 20 and 22 | 2 | Peer research | Facilitation of 6 focus groups and data analysis | Peer researchers are vulnerable and need support and protection. Power imbalance. | Provided de-briefing at end of fgds and team meetings. Had known and trusted support worker present in the building for all meetings, training and discussions. |
| Thulien, M., Anderson, H., Douglas, S., Dykeman, R., Horne, A. et al. | 2022 | To reflect on a CBPR partnership between university researchers and young people with current or past experience with drug use and homelessness in Greater Vancouver. | Reflective | Qualitative | Canada | Drug use & homelessness | 17 to 28 | 10 | CBPR | Youth advisory council (YAC) - work on various research projects. Activities include development of grant proposals, interview guides, community reports, podcasts, videos and events for community audiences. | Complex and diverse positionalities. YAC members have a history of being ignored by workers, providers and other professionals - needed to rebuild confidence in value of collaboration. How to uphold inclusion. Shifts in YAC members life circumstances impact how they contribute to YAC. | Importance of tangible outcomes. Consistently affirm YAC members existing expertise and support development of new skills and confidence. Use a trauma informed approach to foster trust and choice. Minimise barriers for YP who take drugs to be involved e.g. no rules re substance use, number of meetings must attend or what participation should look like. |
| Törrönen, M., Vornanen, R. | 2014 | To bring about change in alternative care arrangements, particularly those involving young people’s transition to independence. | Descriptive | Qualitative | Finland | Care leavers | Average age 22 | 10 | Peer research | 1. Discussion of the themes of the interviews 2. Formulation of the questions 3. Learning the research skills to do the interviews 4. Completing the interviews 5. Sending interview data by email 6. Reflection on the results 7. Dissemination of the results. Attended national and international meetings. | Due to structure of the Finnish child welfare system no contact information readily available for young people who have left care; As co-researchers, the YP were deeply involved in the same experiences as the interviewees, could be problematic if they had had traumatic experiences; Co-researchers interest in the project decreased during the later phases - the YP had passions other than research. | Co-researchers encouraged to follow interview topic guide and not discuss their own experience, did not interview people they knew. |
| Valdez, E., Skobic, I., Valdez, L., O Garcia, D., Korchmaros, J., et al. | 2020 | To identify and describe YPAR studies in the context of youth substance use prevention research. | Review | Systematic review | World | Drug use | NI | N/A | Participatory | N/A | Youth and staff turnover; limited time to complete deliverables; limited resources and budget. Sustainability and achieving long term impact. | Enhanced power sharing and equitable decision-making between researchers and youth. Use of outcome evaluation. |
| van Staa, A., Jedeloo, S., Latour, J. M., Trappenburg, M. J. | 2009 | To evaluate the feasibility, benefits and limitations of a participatory research (PR) project involving chronically ill adolescents as co-researchers. | Reflective | Qualitative | Holland | Chronic illness | 15 to 17 | 9 | Partcipatory | co-developed an interview protocol and during a disco party held for this purpose interviewed each other and 25 fellow patients (12–19 years). They provided advice on the draft report and participated in the dissemination of the results, but were not involved in the design of the project or analysis of results. | Chronically ill young people had low energy levels | The co-researchers felt grateful towards hospital staff and wanted to give something in return. Motivations for being co-researcher included: opportunity to learn interviewing skills; visiting a newspaper office; meeting fellow-patients and giving feedback to hospital staff. Also to earn a little money . |
| Varjavandi, R. | 2017 | To investigate the use of visual methods in a youth-led participatory action research and their potential in exploring resilience-enabling factors in the context of gender inequality that gives rise to the prevalence of sexual violence, teen pregnancy and the blesser phenomenon | Descriptive | Qualitative | South Africa | Low income | 14 -17 | 20 | PAR | Topic identification, develop data collection tool, data collection and interpretation, action plan, implementation, evaluation | NI | NI |
